# Evaluation of a Novel Non-Penetrating Electrode for Use in DNA Vaccination

**DOI:** 10.1371/journal.pone.0019181

**Published:** 2011-04-29

**Authors:** Amy Donate, Domenico Coppola, Yolmari Cruz, Richard Heller

**Affiliations:** 1 College of Medicine, University of South Florida, Tampa, Florida, United States of America; 2 Center for Bioelectrics, Old Dominion University, Norfolk, Virginia, United States of America; 3 College of Health Sciences, Old Dominion University, Norfolk, Virginia, United States of America; 4 H. Lee Moffitt Cancer Center and Research Institute, Tampa, Florida, United States of America; University of California at Berkeley, United States of America

## Abstract

Current progress in the development of vaccines has decreased the incidence of fatal and non-fatal infections and increased longevity. However, new technologies need to be developed to combat an emerging generation of infectious diseases. DNA vaccination has been demonstrated to have great potential for use with a wide variety of diseases. Alone, this technology does not generate a significant immune response for vaccination, but combined with delivery by electroporation (EP), can enhance plasmid expression and immunity. Most EP systems, while effective, can be invasive and painful making them less desirable for use in vaccination. Our lab recently developed a non-invasive electrode known as the multi-electrode array (MEA), which lies flat on the surface of the skin without penetrating the tissue. In this study we evaluated the MEA for its use in DNA vaccination using Hepatitis B virus as the infectious model. We utilized the guinea pig model because their skin is similar in thickness and morphology to humans. The plasmid encoding Hepatitis B surface antigen (HBsAg) was delivered intradermally with the MEA to guinea pig skin. The results show increased protein expression resulting from plasmid delivery using the MEA as compared to injection alone. Within 48 hours of treatment, there was an influx of cellular infiltrate in experimental groups. Humoral responses were also increased significantly in both duration and intensity as compared to injection only groups. While this electrode requires further study, our results suggest that the MEA has potential for use in electrically mediated intradermal DNA vaccination.

## Introduction

The development of vaccines is widely considered to be one of the most important medical advancements of the 20^th^ century. Current methods have been pushed to the limits of their potential. New techniques need to be developed and employed to combat a new generation of diseases and infections. There are several advantages to DNA vaccination. DNA vaccines are cost effective to produce, they can be easily stored, they are highly specific and their multivalent nature means that they could be combined to vaccinate against several different components simultaneously [1]–[3]. Either due to low expression or lack of immune recognition, injection of plasmid DNA alone does not elicit a strong enough immune response for protective vaccination. Electroporation (EP) is a non viral plasmid DNA delivery approach that effectively enhances plasmid expression [4], [5] and immunity [6]–[10].

EP requires the application of electric fields causing permeabilization of the cell membranes. The permeabilized membrane briefly contains “pores” that allow large molecules, like DNA, to enter the cell. Initial studies evaluating *in vivo* EP for transgene delivery and expression were performed on rat brain tumors [5] and rat livers [4]. Those studies demonstrated enhanced delivery and expression of plasmid DNA from EP mediated delivery. Successful EP mediated DNA delivery has been demonstrated in most tissue types and for several therapeutic and prophylactic indications such as cancer therapy, infectious diseases, wound healing, metabolic disorders and vaccines [11]. Recently several clinical trials have been initiated. Two clinical trials have been completed using EP, one assessing tolerability of intramuscular delivery [12], [13] and the other assessing toxicity and clinical utility of delivering pIL-12 intratumorally by EP to melanoma patients [14]. The latter demonstrated the safety, minimal toxicity, and feasibility for the use of EP in the clinic [14]. Since the successful completion of these studies, 19 others are currently active or recruiting. Five of those are involving DNA vaccination against infectious agents (clinicaltrials.gov; Keyword: Electroporation).

Initial *in vivo* EP DNA vaccine studies evaluated gene expression and immune stimulation from delivery of plasmids encoding either Hepatitis B Virus (HBV) protein or Human Immunodeficiency Virus (HIV) protein, gag, to the muscle. Their results confirmed that increased humoral responses to HBV [6] and cellular [9] immune response to HIV gag from EP compared to injection only (IO) of plasmid DNA. More recent studies have broadened the list of pathogens which EP has been successfully used *in vivo* to include other viral pathogens such as: Simian Immunodeficiency Virus [15]–[18], Severe Acute Respiratory Syndrome [19], [20], Influenza [21]–[25], West Nile and Japanese Encephalitis [26], [27], as well as Hepatitis B and C [28]–[32] and Human Papilloma Virus [33], [34]. EP delivered DNA vaccines expressing proteins of the parasitic infection *Plasmodium falciparum*, one of the parasites causing malaria [35], as well as bacterial infections like Bacillus *anthracis* [36], *Clostridium botulinum* [37], and *Mycobacterium tuberculosis* [38] have also been demonstrated to enhance immunogenicity. These results demonstrate the capacity of EP to enhance not only gene delivery and protein expression but also its ability to stimulate the host immune response against a wide variety of pathogens.

Current electrically mediated DNA vaccines employ painful invasive needle electrodes that are inserted into the muscle for electrical stimulation. The primary tissue used for *in vivo* EP is muscle because it is accessible, highly vascularized, multinucleated, and expresses DNA for long periods of time due to the post-mitotic nature of the tissue [39]. However, pain associated with administration is not desirable. As such, alternative delivery sites and methods have been explored. The skin is an attractive target for vaccination because of the high proportion of antigen presenting cells (APC) and large surface area. Recent studies, as well as work done in our laboratory, demonstrated that intradermal electrically mediated DNA expression can be increased both locally and systemically [8], [40]–[44]. Electrodes developed for skin EP include: caliper, plate, tweezer, and clip electrodes as well as several needle electrodes [14], [45]–[48].

**Figure 1:**
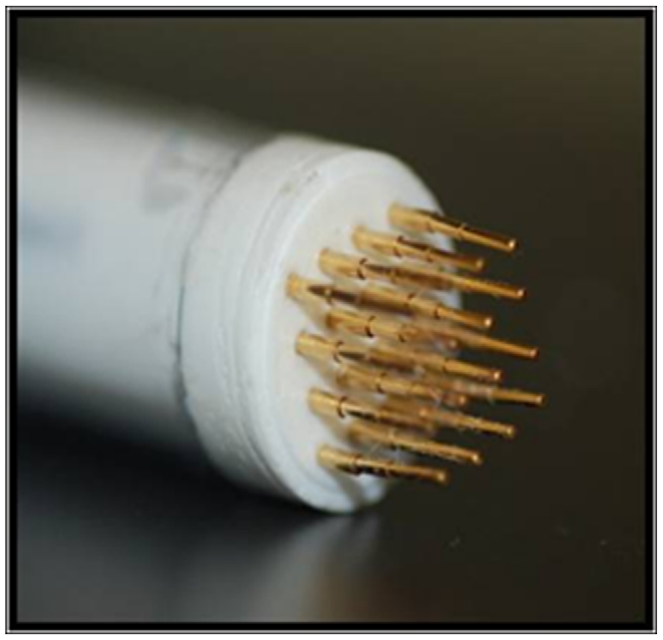
Non-invasive Multi-Electrode Array. The MEA has 16 electrodes placed 2 mm apart and is arranged in 4 rows. Pulses are administered in a sequence that utilizes 4 electrodes at a time, forming 2×2 mm squares (9 total squares). Pulses are applied in pairs, in two directions, perpendicular to each other (18 pulses) for 4 rounds of pulsing (72 total pulses). This image is reprinted from The Journal of Controlled Release doi:10.1016/j.jconrel.2011.01.014 Siqi Guo, Amy Donate, Guarav Basu, Cathryn Lundberg, Loree Heller, Richard Heller “Electro-gene transfer to the skin using a non-invasive multi-electrode array” with permission from Elsevier.

To develop an electrically mediated intradermal DNA vaccine we utilized the non-invasive multi-electrode array (MEA), shown in figure 1, for EP delivery. The MEA has 16 electrodes placed 2 mm apart and is arranged in 4 rows [45]. Pulses are administered in a sequence that utilizes 4 electrodes at a time, forming 2×2 mm squares (9 total squares). Pulses are applied in pairs, in two directions, perpendicular to each other (18 pulses) for 4 rounds of pulsing (72 total pulses). While we have not as yet modeled or directly measured the fields generated across the treated area of skin, we believe by applying the field across a smaller area (2×2 mm) will facilitate obtaining a more uniform field then would be obtained when the field is applied across the entire treated area (6×6 mm). Our lab previously demonstrated that this electrode, when used in a guinea pig skin model, could significantly increase reporter gene activity [45]. Conditions required for optimal expression were determined to be between 200–300 V/cm and 150 ms.

An additional consideration for establishing a MEA delivered DNA vaccine is choosing the appropriate animal model. Guinea pig skin is similar to human skin in thickness and morphology [49]. For this reason, we selected the guinea pig model to better evaluate our delivery approach utilizing a small animal model with skin similar to humans. Therefore, the goal of this study was to evaluate intradermal MEA EP delivery of Hepatitis B surface antigen in a human-like skin model.

## Methods

### 2.1 Ethics Statement

All animal procedures were conducted in a facility (USF) that is fully accredited by the Association for the Assessment and Accreditation of Laboratory Animal Care (AAALAC) and the Public Health Service (PHS). Research was conducted under a protocol approved by the Institutional Animal Care and Use Committee (IACUC) at the University of South Florida, College of Medicine (protocol # 2879). All animals were housed, handled and utilizing following guidelines of the United States National Institutes of Health.

### 2.2 Animals

Female Hartley guinea pigs between 200–250 g were used in this study to evaluate skin EP conditions. Guinea pigs were housed at the University of South Florida, College of Medicine vivarium and were rested for one week prior to experimentation. Guinea pigs were anesthetized with 2.5–3.0% isoflurane before and during all procedures. No previous exposure to Hepatitis B virus was known. *2.2 Plasmid*: The plasmid used in this study was gWiz™ HBsAg (Aldevron, Fargo, ND). This plasmid encodes for the surface antigen of Hepatitis B and is driven by the CMV promoter.

### 2.3 Immunization

All guinea pigs were intradermally injected with 100 µg (2 mg/ml) of gWiz™ HBsAg at two sites on the left flank. MEA EP was performed at 300 V/cm and 150 ms and 72 pulses. The two groups used in this study were control group injection of plasmid only (IO) and injection of plasmid plus EP (I+EP). All groups were boosted with the same condition at Day 14.

### 2.4 serum collection

Guinea pigs were bled through the jugular vein at various time points from Day 0 through Day 168. Blood was collected and serum isolated in serum separator tubes. Serum was diluted two-fold starting at 1∶10.

### 2.5 Tissue collection

Guinea pigs were treated as described with gWiz™ HBsAg with and without EP. Those guinea pigs whose tissue was collected for plasmid expression were sacrificed 48 hours after one treatment and skin samples were harvested by excising the treatment site and snap frozen. Those guinea pigs whose tissue was collected to assess damage and cell infiltrate were treated and harvested 96 hours after one treatment and the tissue was snap frozen.

### 2.6 Indirect ELISA for the detection of Hepatitis B surface antigen antibodies

The enzyme linked immunosorbant assay (ELISA) was used to assess the production of antibodies from treatment and performed per manufacturer's protocol (Aldevron). Briefly, a 96-well plate (Nunc) was coated with 10 µg/ml of Hepatitis B surface antigen (Aldevron) and allowed to coat overnight at 4°C. The plate was blocked with 3% BSA in PBST for 2 hours at 37°C. Serum samples were two-fold diluted in blocking buffer and added to the plate for 2 hours at 37°C. Goat anti-Guinea pig-AP antibody was added at a 1∶10000 dilution in blocking buffer. AP substrate, pNPP, (Sigma) was added to colorize and the plate was read at 405 nm.

### 2.7 Immunohistochemistry

Pathological analysis of the skin sections was performed to determine the extent of plasmid expression as well as inflammation and tissue damage. An anti-HBsAg was used to detect plasmid expression. Skin samples taken 48 hours after treatment were frozen, sectioned, and placed on slides. Slides were rehydrated and then blocked with 3% BSA in PBST and incubated in a humidifying chamber for 1 hr. A HRP conjugated anti-HBsAg (AbD Serotec) was made in blocking buffer at a 1∶200 dilution. All samples were counterstained with Hematoxylin & Eosin. Samples collected at 96 hours frozen, sectioned, and placed on slides were stained with H & E to determine the extent of cellular infiltrate/inflammation.

### 2.8 Statistical analysis

All Guinea pigs were bled at Day 0 to determine background optical density (OD). OD's were averaged and 2 standard deviations added to determine positive (0.1 OD). Experimental serum samples were diluted two-fold starting at 1∶10. End point titers were calculated and plotted as Geometric Means. Significance was determined by student t-test using the bonferroni correction for multiple comparisons.

## Results

### 3.1 Plasmid expression from EP

The first step in evaluating the MEA for delivery of DNA vaccines in a human-like model was to determine the expression levels of gWiz™ HBsAg. Guinea pigs were treated as described with or without EP using the MEA. 48 hours after delivery the guinea pigs were euthanized and the treated skin harvested and processed for histological evaluation. Expression of HBsAg was determined by immunohistochemistry. Expression of HBsAg is seen in IO and I+EP (Fig. 1a and b), however increased staining was observed in the I+EP samples. Expression is contained within the epidermis of IO animals. When compared to I+EP animals expression can be seen within the epidermis and dermis.

### 3.2 Immune cell infiltrate and tissue damage

**Figure 2:**
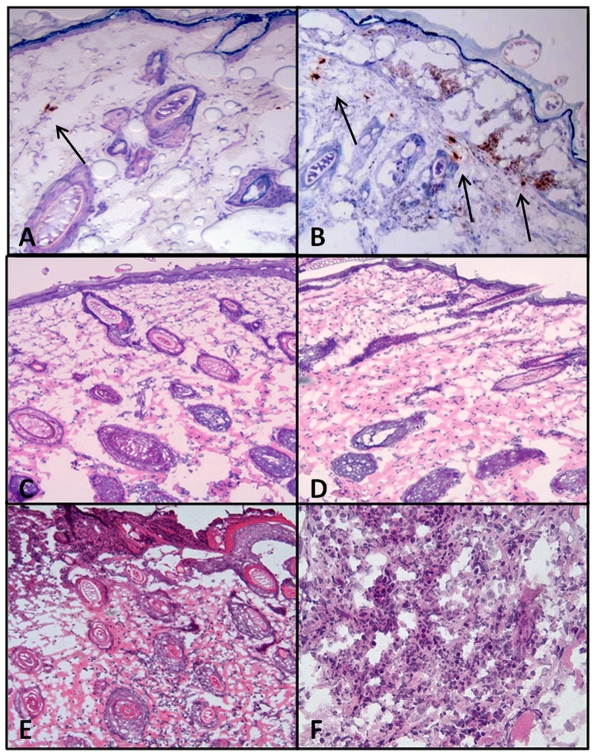
Plasmid expression and inflammation in the skin. Guinea pigs were treated as described in Methods 2.1 with pHBsAg. Expression of plasmid was evaluated at 48 hrs post treatment by IHC (A-IO; B I+EP). Inflammation was measured 96 hrs post treatment and assessed by H&E (C-No treatment; D- IO, E–I+EP) at 100× magnification and 200× magnification (F- I+EP).

To determine whether EP with the MEA would recruit immune cells to the treatment site and cause inflammation, guinea pigs were treated as described and tissue samples harvested 96 hours after treatment. Samples were stained with H&E to assess cellular infiltrate, damage, and necrosis from treatment. The induction of immune stimulation is important for vaccines in general, but can be limited for DNA vaccines. Induction of immune cell infiltrate was observed (Fig. 2 C–F 100× magnification). Background levels, Fig. 2c, of infiltrate are demonstrated in no treatment control and correspond to low levels of cellular infiltrate (purple). IO samples show slight increases in infiltrate as compared to no treatment, Fig. 2d. In contrast, I+EP samples show a large increase in cellular infiltrate, Fig. 2e. I+EP groups contained primarily macrophages and multi-lobed cells, most likely activated neutrophils (200× magnification Fig. 2f), corresponding to a prolonged inflammatory immune response [50].

**Figure 3:**
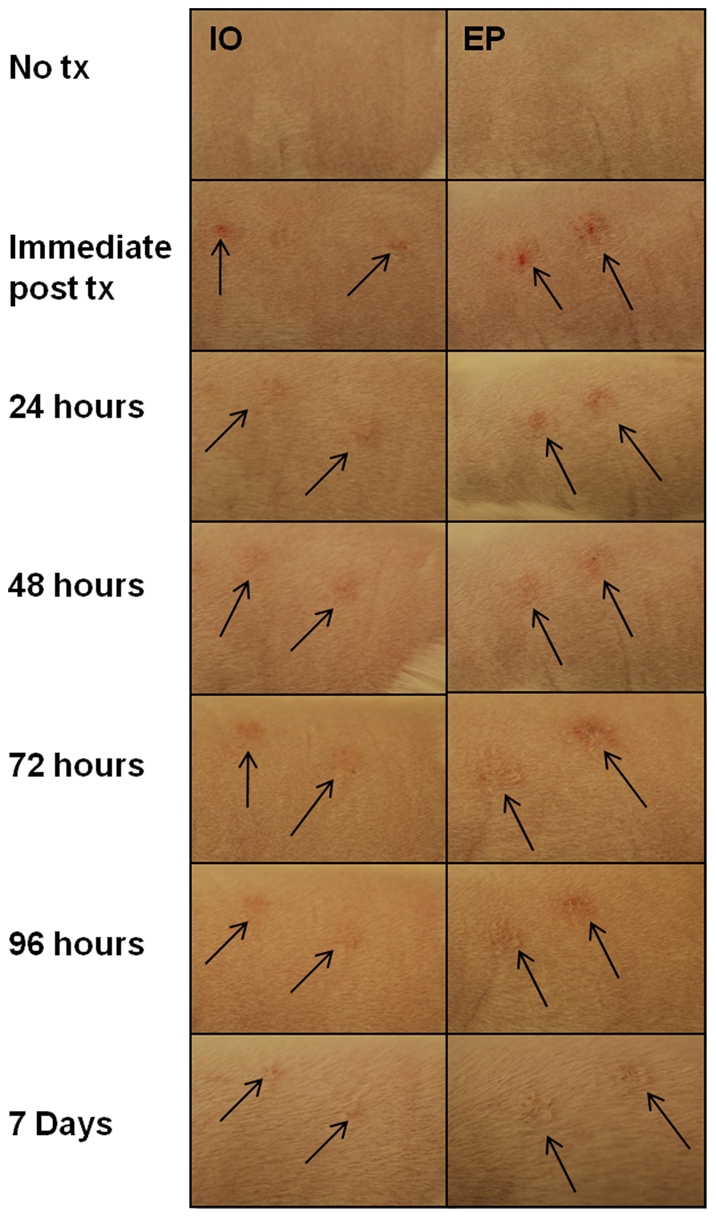
Visual assessment of skin damage and healing. Guinea pigs were treated as described in Methods 2.1 with pHBsAg with or without EP. Images were taken of skin pre treatment, immediately post treatment, and at 24, 48, 72, 96 hours and at 7 days. Arrows indicate the treatment sites.

Edema was seen in all samples except no treatment controls; and did not appear increased due to EP. This is most likely a result from the injection of plasmid into the tissue. In most samples tissue damage and necrosis were not seen. However, two EP delivered samples had minimal ulcerations at 96 hours after treatment, one of which also had about 1% necrosis. There were no other samples showing damage or necrosis (data not shown). Gross evaluation of the skin shows no difference between IO and I+EP groups over time (Fig. 3). Complete visual recovery of the skin is seen by Day 7.

### 3.3 Anti-Hepatitis B surface antigen antibodies

**Figure 4:**
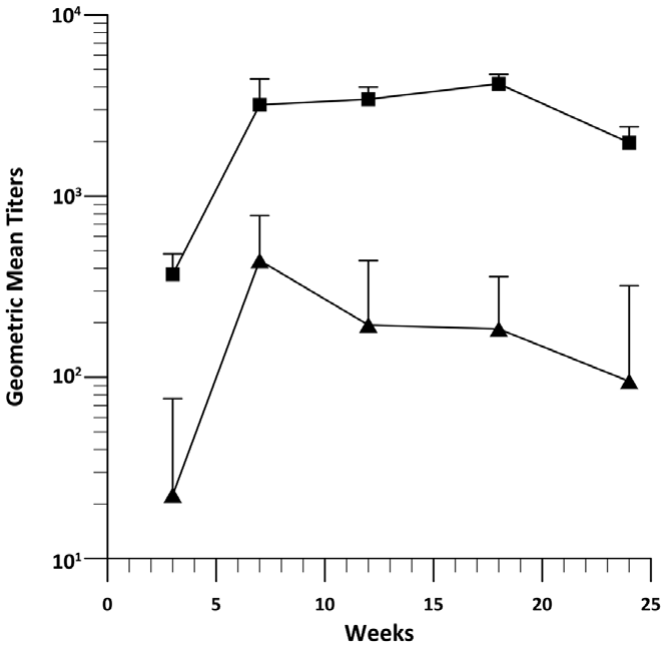
Evaluation of anti-HBs serum titer. Guinea pigs were treated as described in Methods 2.1 with pHBsAg. Serum was collected at multiple time points and an ELISA performed. Geometric mean titers are expressed. Positive was determined by two standard deviations greater than the Day 0 OD. IO and EP n = 6 for each experiment with 3 independent experiments conducted (total n = 18). Statistics were determined by two-sided student t-test with bonferroni correction to p<0.05.

While cellular infiltrate can be an early indicator of immunity, a more accurate measure is the induction of specific antibodies generated against HBsAg. Anti-HBs were measured by ELISA over time. Guinea pigs, treated and serum collected as described in methods, showed significant increases in antibody expression from three weeks after initial treatment through week 24. The data collected was from 3 independent experiments (n = 6 for each experiement) with a total n of 18 for both IO and EP groups. Peak expression for both groups occurred at week 18 with IO groups having a GMT of 1000 and I+EP animals at 5000 (Fig. 4). The fold increase over IO remained relatively constant at about 5 fold with the greatest fold increase over IO of 6.5 occurring at week 18.

## Discussion

These data demonstrate that the MEA can be effective for the use in electrically mediated DNA vaccination in a human-like skin model. EP with the MEA generated increased plasmid expression as well as an increase in immune infiltrate after treatment. The magnitude of immune infiltrate was greater in EP groups than IO and there was minimal to no skin damage associated. Specific, lasting, and significant levels of antibodies were greater than IO. This is the first report to demonstrate the use of the MEA for DNA vaccination in a human-like skin model.

DNA vaccination is advantageous because it does not integrate into the host DNA, it is cost effective to produce and easily stored, it can be highly specific for tissue and/or cell type and can be made to vaccinate against multiple agents simultaneously. The skin is an ideal target for DNA vaccination due to the large surface area and presence of antigen presenting cells like langerhan's and dermal dendritic cells, specialized for induction of immunity [51].

However, injection of plasmid alone does not induce high enough immune responses to be protective. EP is one method that has been shown to increase both plasmid expression as well as immunity. Previous EP methods have involved painful penetrating electrodes that go into the muscle to facilitate delivery. Further advancements have been made using non-penetrating electrodes such as caliper and plate electrodes. However, these electrodes require high voltages to enhance delivery and therefore can cause tissue damage. In this study, we have evaluated a non-penetrating electrode which reduces the gap width between electrodes to 2 mm thereby reducing the absolute voltage applied and preventing visible tissue damage while still increasing plasmid expression and immunity.

As expected from our previous publication [45], EP with the MEA enhanced expression. While the exact reason for the effectiveness of EP remains unknown, increased plasmid expression at least in the case of DNA vaccination, plays an important role in recognition by the immune system [52]. EP has been shown to have an adjuvant effect by recruiting immune cells to the site of pulse application [53]. In our study, we saw an influx of nucleated cells from EP treated samples. These cells are most likely neutrophils and macrophages based on morphology. This is most likely a combination of both an EP mediated adjuvant effect and increased plasmid expression. The induction of macrophages and polymorpho-nucleated neutrophils is indicative of a chronic inflammatory response. While the perception of prolonged inflammation is typically negative in our case it indicates that the expression of the plasmid is present for a prolonged period of time, giving the immune response enough time to perform its function. Based on our earlier work [45] we would expect this prolonged expression to decrease after approximately 14 days, therefore allowing the body to heal and not generate deleterious effects from inflammation.

These findings seem to correlate with our antibody data, where an increase in the presence of specific antibodies was measured over time. These antibodies were significantly increased as compared to injection only. Geometric mean titers ranged from 4000–16000 peaking at week 18. Antibody levels remained elevated until dropping off after week 21, but still remained increased as compared to injection only. The enhanced intensity of humoral immunity by EP with the MEA corresponds to previously published skin EP results [54]–[57]. One of the primary reasons for evaluating our delivery method with Hepatitis B was because it is a well characterized vaccination model. Published studies have reported geometric mean titers in conjunction with protective efficacy in guinea pigs. While the presented GMT's in these papers were higher than ours, they also reported protective levels more than 100 fold above the necessary levels. Our GMT's are likely to still be within the protective range without generating unnecessary additional responses [58], [59]. Comparing specifically to Hepatitis B DNA vaccines delivered by EP several animal models have been evaluated and EP has been shown to have protective levels from 10–1000 mIU/ml [6], [9], [32], [60], [61]. The most recent comparable publication evaluated a minimally invasive device for protective vaccination against influenza [62]. While their results were only presented as neutralizing titers against flu and cannot be compared directly we believe that our electrode design generates immune responses of equal quality without tissue penetration.

The data represented here demonstrate the capability of the MEA to increase plasmid expression, immune cell infiltrate and inflammatory response, as well as antibody production over 24 weeks in a human-like skin model. This information presents a potential new method for DNA vaccination that may be translatable to humans. Further studies will examine the MEA for use in DNA vaccination against other infectious agents.
